# Book Reviews

**Published:** 1814-12

**Authors:** 


### CRITICAL ANALYSIS

### *OF RECENT PUBLICATIONS*

### IN THE

### DIFFERENT BRANCHES OF PHYSIC, SURGERY, AND MEDICAL PHILOSOPHY.

*Facts and Observations relative to the Fever commonly called Puerperal.* By John Armstrong, M. D. Member Extraordinary of the Royal Medical Society of Edinburgh, and one of the Physicians to the Sunderland Dispensary.—8vo.; pp. 162; Longman and Co. 1814.

THE subject of this treatise is perhaps the most important that can come before a practitioner. The rapidity with which the disease hastens to its termination, and the fatality of that termination when unassisted by art, are well known, as well as are the symptoms which usually announce puerperal fever. But, although there may not be so much difference of opinion respecting the nature of this disease as the author supposes, and seldom any hesitation in discriminating it from other complaints, considerable difference has prevailed in the treatment, and that amongst practitioners of such judgment and experienced tact, that there is still great room for a work in which the subject may be fairly canvassed, and the question set at rest for ever. We conceive the publication before us, although small and unostentatious, to have materially contributed towards this end, and, as far as our own experience extends, we can unite in opinion with the author in most of the points which he has advanced. We say in most of them, because there are some on which we cannot decide so positively: we have not found it necessary to give purgatives quite to the extent which he would recommend, viz. half a dram of calomel for a dose, &c.; we do not agree with him that there is no difference between puerperal fever and peritonitis; neither do we think that it is always contagious.

 The

The opportunities which Dr. Armstrong has enjoyed of witnessing the disease have been considerable; yet he has not been equally successful in obtaining an examination of the body after death in such cases as terminated fatally. From what he has observed, however, he is decidedly of opinion that the complaint in its origin is local. Can this opinion accord with his conviction that the disease is also contagious? that it can be communicated from one lying-in woman to another, simply by a nurse or accoucheur visiting a sound person in another house, after having attended a patient in puerperal fever?

The complaint from which he has chiefly furnished the present observations, in Sunderland and its neighbourhood, "generally occurred about twenty-four or thirty hours, and seldom later than four days, after delivery. It did not seem to depend upon difficulty of labour, for in most of the women in whom it occurred parturition was remarkably easy, and the placenta was cast off after a proper interval, and without more than usual pain. Nor was the lochial discharge, before the attack, in any way apparently affected. The disease was ushered in by very slight shiverings, or rigors, by oppression at the præcordia, by vomiting, retching, or nausea, and by considerable anxiety of mind. When the shiverings or rigors abated, which were often very short, the skin became universally hot and dry, and the thirst urgent. The tongue was much paler than usual, and appeared as if it had been recently rubbed, or dusted with a very fine whitish powder; in some few instances, however, the tongue was tolerably clean and moist about the edges, and this was more especially the case when vomiting frequently occurred. The matter thrown up consisted of the ingesta, mixed with mucus, and yellow or greenish bile. The pulse was seldom less than 120 in the minute, and rather full, tense, and vibrating, or very small, sharp, or somewhat wiry.

"The countenance at this period assumed an inexpressible anxiety, the lips were pale and parched, and there was a kind of livid stripe under each eye, but the cheeks were flushed with a circumscribed redness, like that which is observed in the true hectic. The respiration soon became hurried, and the patient often sighed heavily, was restless, and turned from one part of the bed to another, or lay upon her back, and constantly moved her head from side to side, or suddenly lifted up her hands, or threw them down again with some force upon the bed-clothes. Commonly a little before, or at the very commencement of the shiverings or rigors, there was in the lower part of the belly more or less pain; occasionally it was very acute, shooting in the direction of Poupart's ligament, and through to the back and loins. In some instances, the pain was deep and obtuse, and more confined to one particular part; but in every case, it was aggravated by pressure in and about the hypogastric region. However limited in its extent at first, it afterwards gradually spread over the surface of the abdomen, which became tender to the touch, tumid, and tense."

The author only noticed in three cases the enlargement of the abdomen mentioned by Dr. John Clarke in his dissertation on the low epidemic of lying-in women; but it occurred frequently in the practice of Mr. Gregson, a surgeon of Sunderland. He also observed in several of the cases substances resembling hard bands or chords, which passed completely across the abdomen, and might be distinctly traced beneath the muscles by moderate pressure of the finger.

"The secretion of the milk was nearly suspended soon after the attack, the breasts became flaccid, and the mother, so lately all solicitude about her child, now seldom inquired after it, and indeed seemed almost insensible to those things which before most deeply interested her feelings. The lochial discharge either disappeared or only issued in small quantity, and was very dark and uncommonly offensive. The urine was scanty and high-coloured, but generally passed without much pain. The bowels were constipated and flatulent, and in two instances something similar to the globus hystericus was observed. Though all the patients were restless in the extreme, seldom obtaining a moment's sleep, yet they never complained of violent pain in the head, but of an uncomfortable aching and lightness there. The eyes, when the fever was at its acmé, seemed rather brighter than natural, and the pupils were slightly dilated. The whole train of symptoms already described may, in a practical view, be called the first stage of the disease—the stage in which alone a fair opportunity is offered to the practitioner of saving the life of the patient.

"This state of febrile excitement, in most of the cases which lately occurred, seldom continued longer than fifty hours, and in some it terminated much sooner. When the disease was not impeded at this period, it passed into what may be termed the second and last stage, which, toward the close, was marked by an exceedingly great prostration of all the vital powers.

"But the first approaches of this fatal stage were most clearly indicated by the rising of the pulse, which then generally ranged from between 140 and 160 in the minute, and was very soft and compressible; it feebly struck the sides of the artery, and gave the idea that the heart was labouring hard to keep up the force of the circulation. About twelve hours before death, the pulse became thready, fluttering, and irregular, and so rapid as not to be correctly numbered.

"For some time after the accession of the second stage, the skin remained at an increased temperature, and dry, but then the patients almost constantly complained of chilliness. The cheeks were alternately flushed and deadly pale, the eyes lost their lustre, the pupils were much dilated, and a kind of dewy perspiration stood upon the face and forehead. The pain gradually and entirely receded from the surface of the abdomen, when it usually happened that dark, slimy, and very fetid stools were discharged from that time onward. The thirst was unceasing, and when any liquid liquid was offered, the patients hastily seized the vessel, and glutted down its contents, as if they had previously been expiring for want of drink. The tongue for the most part was brown, or rather black and parched, and had apthæ upon it, which even appeared about the edges of it at an early period. In one very bad case, however, the tongue continued clean and moist to the last, but there was an almost perpetual vomiting throughout the second stage, though only a slight nausea occurred in the beginning, and very little vomiting in the rest of the first stage. Indeed vomiting was always more urgent in the last than in the first stage of the disease, and the matter then thrown up very much resembled coffee-grounds, and was offensive to the smell. The teeth and gums were crusted with dark slimy sordes, and the breath was disagreeable, as if it had been tainted with mercury. Throughout the complaint there was a short teazing cough, but this was more especially the case in the last stage, when the respiration grew very short, feeble, and frequent, and the alæ nasi were thrown into perpetual motion.

"Soon after the advancement of the second stage, the patients began to talk incoherently; they frequently made attempts to get out of bed, and occasionally, after having laid still a short time, suddenly started, and spread out their hands, which were then very tremulous, as if to ward something off that was approaching them. About this time, two patients became gradually collected, complained of no pain whatever, looked and spoke cheerfully, and flattered themselves that they would soon be well: this illusion continued till within an hour or two of their departure, rendering them completely insensible to their real situation; and even to friends, though warned by the medical attendant, their death was at last unexpectedly sudden. But in three other unfavourable cases, the light wanderings of the mind which took place at an early period of the second stage, were not succeeded by a state of serenity, but by a low muttering delirium, speedily followed by a stupor, in which the patients lay with their eyes half-closed, and could not be roused from it, but by loud speaking, upon which they started as from a disturbed sleep, uttered some vague and hasty expressions, and then sunk into the same condition as before. A few hours before death, in these cases, some dark scattered petechiæ appeared, and the skin was in that peculiar state which accompanies the last stages of tetanus, and the nervous fever of intoxication; the whole surface felt soft, relaxed, and clammy, and the hand glided almost as smoothly over it as if wet by soap and water. In the above three instances, also slight stertorous breathing occurred near the termination of the disease, and, last of all, general, though not violent, convulsions."

In that part of his work which treats of diagnosis, the author again expresses his belief in the similarity, if not identity, of peritonitis with puerperal fever, and considers it essential only to distinguish puerperal fever from "milk fever, after-pains, inflammation of of the uterus, and that ephemeral called the weed, to which child-bed women are very liable." Without being "admirers of nosological minuteness," and without allowing our belief in the two diseases being different, to influence our practice, we still assert the difference. The practice would not be essentially different in peripneumonia, pleuritis, or enteritis; yet the author, we doubt not, will willingly admit that there is a difference between those diseases. It is sufficient for us at present to observe that peritonitis attacks males as well as females, and in these it is not confined to those in the puerperal state, neither do we recollect in our own practice, nor in authors, any instance of peritonitis being contagious. This portion of the treatise, as well as the few pages on the prognosis, contains some good practical remarks, which deserve the attention of young practitioners. The observations on "prevention" may also be perused with advantage, even by those who do not believe that the fever "is *always* infectious," for, as Mr. Hey has prudently observed, though we may not be decided that the disease is contagious, it is at least proper to act as if it was.

Upon the treatment, we entirely agree with the author in principle. To consider the first stage of the disease inflammatory, and to attempt to reduce it by active and powerfully depleting remedies, of which the most efficacious are bleeding and purgatives. If these are had recourse to in the commencement of the complaint, they will prove successful. When the contrary is the case, it will generally be found that the remedies have been too long deferred, or have not been applied with a bold and decided hand. The author recommends that the quantity of blood to be drawn from the arm at once should seldom be less than twenty-four or exceed thirty ounces. That a repetition of bleeding should be avoided, but, if indispensible, a smaller quantity, not exceeding twelve ounces, should be taken, after a short interval. The cathartic medicines, which he at the same time directs, are very powerful, as calomel in doses of a scruple and half a dram at the beginning, and repeated occasionally in the course of the disease. This mode of treatment was successfully pursued by five practitioners in Sunderland, who, out of forty-three distinctly-marked cases of puerperal fever, lost only five.

"The thirty-eight successful cases were all treated by copious depletions of one kind or other; and in twenty-nine of them, calomel was exhibited in doses of a scruple or half a dram at the beginning, and occasionally repeated in the course of the distemper. For the most part, it passed so expeditiously along the intestinal canal, that there were very few instances in which ptyalism was excited, and, whenever this was the case, it seemed a favourable circumstance, all the patients, with only one exception, recovering with more than ordinary celerity from the time that the mouth became affected. And further, to illustrate the superior efficacy of large doses of calomel, it may be here remarked, that in none of the the five cases which proved fatal, more than fourteen grains of calomel were given on the accession of the fever; jalap, sulphate of magnesia, and castor oil, being the cathartics chiefly employed during its progress."

Mr. Gregson, whose name is mentioned before, in addition to this plan, when the patients had been freely bled and purged, prescribed antimonial emetics with decided advantage. Another practitioner, however, Mr. Wolfe, of Chester-le-street, and who has had great experience in puerperal fever, it seems, trusts to purgatives alone, and has been very successful in the cure of the complaint. In a letter addressed to the author, Mr. Wolfe states that, during the last sixteen years, he has always trusted to very copious purging for the cure of puerperal fever, keeping the bowels very open for several days successively; and, in some severe cases, even for two or three weeks before the symptoms of the abdominal inflammation completely subsided. The stools, he observes, are of a peculiar nature, and constitute one of the characteristic marks of the disease: they are of a dark brown colour, resembling coffee-grounds, very copious, of the consistence of thick gruel, and with a fetid smell; it was not till towards the end of the disease, as they were approaching the natural state, that hard scybalæ were frequently discharged, even after a long and active purging had been kept up.

The treatment hitherto has been adapted to the first or inflammatory stage of the disease; when that has passed, and symptoms of a malignant nature, as detailed with great accuracy by our author, succeed, bleeding of course cannot be practised; but throughout the whole course of the disorder, whatever other remedies are employed, purgatives are often indicated, and sometimes are indispensibly necessary. The author, in the worst form of the disease, has also found the strength of the patient better supported by milk and broths, than by wine and cordials, which speedily destroy the patient. It is sometimes necessary to give anodynes.

In an appendix, the author has adduced evidence from other practitioners in favour of his plan of treatment, and has presented us with some interesting cases, in which the efficacy of bleeding and cathartics is abundantly proved. One case, in which the plan had only been partially carried into effect at the commencement of the disorder, is curious, as it evidently demonstrates the ill consequences of half-measures in the treatment of this rapid and destructive malady.

We cannot conclude without strongly recommending Dr. Armstrong's Treatise on Puerperal Fever to the attention of our professional brethren, and hope that he will shortly fulfil his promise of publishing his further practical illustration of fever, having in the tract before us so fully demonstrated his competency to the undertaking.

*Pathological*

*Pathological Researches.—Essay I. On Malformations of the Human Heart: illustrated by numerous Cases, and five Plates, containing fourteen Figures, and preceded by some Observations on the Method of improving the Diagnostic Part of Medicine.* By J. R. Farre, M.D. 8vo. pp. 46.

On a former occasion, in reviewing Dr. Farre's first fasciculus on the "*Morbid Anatomy of the Liver*," we had much gratification in stating his claims to professional notice. To industry in collecting materials, he happily unites skill in arranging them, and it is fortunate for the profession that his philosophic conclusions afford indubitable evidence of the correctness of his judgment. His mind, indeed, seems peculiarly adapted to the pursuit in which he has so successfully engaged. If, in all the weighty affairs of life, in religion and politics, in those great questions which immediately involve and influence the present and future well-being of man, the attractive and rapturous power of imagination yields to the sober unadorned exercise of reason, it is especially so in those inquiries which concern his health and physical organization. The philosopher smiles at the hypotheses and conceited opinions which ingenious men hazard on subjects, where, from the difficulty or the mystery of the inquiry, conjecture may be tolerable; but he regards with admiration the slow, yet regular, advance towards perfection which a steady exploration of facts necessarily causes. To say that the anatomy of diseased parts in human structure is yet behind the progress of other branches of the healing art, is a truism, and, as such, we do not expect to hear it denied. But is this owing to any impossibility in the research, or to indolence and incapacity in practitioners? We conceive partly to the latter cause. From what we have seen effected by Morgagni, by Baillie, and by some even of more recent date, in fact by most of the teachers of anatomy, to say nothing of detached communications in the memoirs of societies and periodical publications, the only difficulty that occurs to us, is that of perseverance, cool application, and sound judgment in the practitioner who undertakes the inquiry, with sufficient zeal and adequate leisure to record the facts which he has ascertained. Simple, however, as some individuals may estimate these qualities, they have rarely existed together in persons who have possessed the opportunity and the power of prosecuting the inquiries in which Dr. Farre is now engaged.

The present essay affords a pleasing testimony of the progress of diagnosis, founded on morbid anatomy, within the last thirty years; in fact, the chief utility of this curious science consists in facilitating our accuracy in distinguishing the signs of disease depending on imperfect organization.

The author divides the imperfections of structure of the heart into two species, viz. I. Malformations of the heart, or of its arteries, mingling black with red blood. II. Malformations of the the heart, or of its arteries, only impeding the circulation of the blood.

The varieties of these we shall notice in the order in which the author has arranged them.

"I. 1. *Single Heart.*"—This variety is rare. The author does not give a case of it.

"I. 1. *a. Two Pulmonary Branches from the Aorta.*—E., a full-sized infant, was born at three o'clock in the afternoon, March 30th, 1807. For the space of half an hour there seemed to be a difficulty in establishing the circulation through the lungs. His respiration was uneasy, and accumulated mucus in the larynx distressed him; his face was for some time very pallid, and afterwards slightly livid. Finally, the important functions of circulation and respiration were performed with freedom. During the first forty-eight hours, he seemed to enjoy the most perfect health: his countenance was lively and ruddy, his skin warm, the meconium and urine were properly evacuated; he took the breast eagerly, and slept easily. On the night of April 1st, his nurse consulted me for a difficulty in his breathing. His respiration, indeed, was remarkably quick, but the temperature and aspect of his skin were natural, and he seemed free from pain. He was undressed to admit of a more particular examination. The action of the diaphragm was unusual, and at each contraction, which was very frequent, it forcibly bent inwards the margin of the thorax. The pulsations of the heart were too strong. Nothing was done, because the indications were not clear. He slept quietly the greater part of the night, but, at an early hour the following morning, I was called to him. His cries expressed the distress he then suffered. The diaphragm laboured excessively, and the whole line of its attachment was marked by its vehement action; the heart thumped against the ribs, the pulse at the wrists could not be felt, the skin was pallid and cold. He was immersed in warm water until his cheeks flushed, and then wrapped in flannel. The circulation on the surface was restored, and his distress was mitigated. It appeared that his sufferings had been increased by a distention of the stomach; for after his mother's milk began to flow, at each time that he sucked, his distress became greater, especially the last time, which immediately preceded the violent exacerbation just described. After the use of the warm bath, his skin never became pallid as before, but remained somewhat cold, for the blood lingered in the cutaneous vessels, and his countenance was slightly livid. His muscular powers faded, and his limbs fell. Previous to the disturbed state of respiration, his strength had been unusually great for that of an infant, but now he had not power even to embrace the nipple, although he would make the effort. The labour of the diaphragm ceased, his respiration became more and more feeble, his sensorium during the last few hours was torpid, but he died without convulsion. His death happened seventy-nine hours after birth, and about thirty after the respiration was affected.

"*Dissection*,

"*Dissection.*—The heart, situated naturally, was distended in the utmost degree with blood. Blood was extravasated under the pericardium, and very extensively into the cellular texture of the lungs, but there was no effusion into the cavities of the chest. The heart consisted of an auricle, a ventricle, and a single artery. The auricle was divided from its appendix more distinctly than it is in the natural structure, by an intermediate septum, which, however, gave a free passage to the blood through a large central aperture. The venæ cavæ opened into the auricle, and the four pulmonary veins into the appendix. There was only one ostium ventriculi. The ventricle was single, and had a valve, which, although it was neither tricuspidal nor mitral, more nearly resembled the former than the latter. From the ventricle, one artery, the aorta, furnished with semilunar valves, arose. Its two first branches were pulmonary, very large, and situated close to each other. The third branch was still larger; it came off at a right angle from the aorta, and gave origin to the arteria innominata, the left carotid, and left subclavian arteries. It also sent down a single artery to the heart, which served instead of the coronary arteries. The continuation of the trunk had the usual appearance of the descending aorta. At the origin of the pulmonary branches, the aorta was considerably contracted in its diameter; its coats were separated by blood which had been extravasated between them from the vasa vasorum, and were also thickened; the inflammation extended along its internal coat towards the heart, and its valves were excessively red. All this was evidently recent, and probably caused by the increased current of blood through the pulmonary branches, the distention of which seemed to have impeded the circulation through the aorta, by making a kind of valvular process at its base. In consequence of such obstruction, too much blood was sent through the pulmonary branches, more was returned to the heart than it could eject, the artery inflamed at the obstructed part, the circulation was thus more and more impeded, till the cellular texture of the lungs was filled with blood, and the overloaded heart ceased to act.

"The abdominal viscera were in a natural condition; the brain was not examined."

This case is illustrated by a neat plate containing three figures.

"I. 1. *b. One Pulmonary Branch from the Aorta.*"—This case is related by Mr. Standert in Phil. Trans. vol. xcv. p. 228, and the heart is preserved in the museum of Dr. Ramsbotham.

"I. 1. *c. Heart transposed. The Aorta and Pulmonary Artery branching from a common Trunk.*"—An account of this very interesting case, illustrated by figures, is communicated by Mr. Wilson in Phil. Trans. vol. lxxxviii.

"I. 2. *Imperfect Double Heart.*"

"I. 2. *a. Unclosed Foramen Ovale.*"—This variety of malformation is not rare, nor always attended with the inconvenience, distress, and fatality, which usually denote imperfect structure of the organ. The author dissents from the opinion of Mr. Allan

 Burns

Burns that it is productive of dyspnœa. Dyspnœa frequently accompanies old age; but some persons in advanced age, who have died of some other complaint, and in whom the foramen ovale after death has been found pervious, were not affected with dyspnœa.

"I. 2. *b. Dilated Foramen Ovale, or Imperfect Septum Auricularum.*"—The author considers this variety rare, and does not appear to have seen an instance of it.

"I. 2. *c. Dilated Foramen Ovale, with an open Ductus Arteriosus.*"—A case of this affection is described by Mr. Spry, in vol. vi. of the Memoirs of the Medical Society of London; and by Mr. A. Burns, in a work on Diseases of the Heart. Dr. Farre also states the following interesting fact. "On the 4th of November, 1813, Mr. English favoured me with an opportunity of examining a case of this malformation. We found the heart of its proper size and figure, its respective cavities being only proportioned to each other; but the valve of the foramen ovale was so very imperfect, that a free communication between the auricles existed. The ductus arteriosus was open, and larger than natural. The pulmonary artery was proportionally larger, but its right and left branches were of their proper size. The liquor pericardii was increased, the other cavities of the chest were free from serum, and the appearance of the lungs was natural. In the abdomen we found the liver overcharged with blood, its gall-bladder, cystic, hepatic, and common ducts, preternaturally contracted; but no other morbid appearance."

Mr. English's statement of the previous history of the case, as related by Dr. Farre, is as follows.

"Mrs. S. was delivered of a girl, on the 15th of October, 1813. At her birth nothing very remarkable was observed. She cried faintly for a few minutes, seemed rather weak, and her skin and eyes were somewhat yellow. The bowels were freely opened by castor oil. An occasional threatening of suffocation when she sucked, unusual quietness, and perpetual drowsiness, were remarked during the first week. In the course of the second week, she had fits of crying, generally in the evening, and her breathing was very remarkable. She would take a deep and long inspiration, sobbing once or twice during the time, and afterwards breathe very quick. Presently, the inspirations would be short, and the expirations uncommonly long, until she was roused with a sort of convulsive sob, attended with a slight crowing noise. This peculiarity in the respiration was manifested chiefly after sucking or crying. The paroxysm being over, she would fall asleep, and breathe easily, perhaps more quickly than natural, but start frequently. On the fourteenth night the fit of crying was more severe than on any former occasion, and for several moments the breathing was suspended, the lips becoming black, when a strong convulsive effort with a deep sigh restored animation. This took place repeatedly for about an hour, but afterwards the night was passed quietly. On the fifteenth and sixteenth days, the breathing became more and more disturbed; but the evening paroxysm of the seventeenth seventeenth was thought to be somewhat less violent than the preceding ones, and the night was passed more comfortably. On the eighteenth morning the child took the breast with less suffocation than usual. The skin and eyes remaining very yellow, and the fæces deficient in bile, a grain of the submuriate of mercury, and four grains of rhubarb, were given at four o'clock in the afternoon. The child slept about an hour, awoke, and vomited a part of the powder, was soon afterwards apparently in much pain, and screamed violently. In this paroxysm, the breathing was frequently suspended for more than a minute, the lips were black, and the eyes fixed. A medical gentleman in the neighbourhood administered a dose of castor oil, some assafœtida, &c. but, at eight o'clock, when Mr. English saw her, she appeared to be dying. The extremities were cold, the countenance cadaverous, the lips black, the breathing by short convulsive inspirations and long groaning expirations, with now and then a sigh. On putting her into a warm bath, it was surprising to see the improved state of the respiration; the expirations being assisted by gentle pressure on the abdomen and ribs. This treatment having been continued for half an hour, she was wrapped in warm flannel, and seemed to sleep for an hour, when the breathing gradually got worse, and all the former train of symptoms returned. The warm bath was again resorted to, with the same benefit as before; but, on taking the child out of the bath, she soon ceased to breathe if left to her own efforts. He therefore kept his hands constantly on the thorax and abdomen, and by assisting the expirations, prolonged her life for about two hours. For ten minutes together she sometimes lay without the least appearance of life, when a strong convulsive action of all the muscles of the thorax and abdomen, with a deep sigh, and several catching sobs, renewed the circulation. Then the colour would return to her lips; she would stretch her limbs, and open her eyes. The last struggle of this kind took place a quarter of an hour after he supposed that she was dead. He never could feel the pulsations of the heart, although he frequently examined the region of that organ. The pulsations of the radial arteries, he once observed, were synchronous."

(To be continued.)

---

*Memoire sur le Vomissement.* Par M. MAINGAULT, Docteur en Medicine, &c. pp. 20.

MR. MAGENDIE's Experiments on Vomiting have been already made known through the medium of this Journal. Surprised to observe they deprived the stomach of the power of contraction during that action, M. Maingault has undertaken a series of curious experiments, which are the object of the present memoir, and the results of which differ essentially from those obtained by the ingenious physiologist in question.

The experiments of M. Maingault seem at first to prove that vomiting takes place independently of the abdominal muscles and of of the diaphragm, organs which, according to Mr. Magendie, are the sole agents of this phenomenon. We shall not at present decide how far apparently contradictory facts of the two physiologists are capable of being reconciled; but the labours of M. Maingault will be received with pleasure by those who are really interested in the progress of physiological science.

Our author having opened the belly of a dog, and removed the abdominal muscles, produced strangulation of the intestine, and the animal vomited several times. In other experiments, *the diaphragmatic nerves having been divided*, and the strangulation of the intestine having been effected through a little opening in the abdomen, the vomiting took place. In other dogs, the author divided the diaphragmatic nerves, made the section of the abdominal muscles, and removed a portion of the diaphragm *(jusqu'au centre phrenique)*. He then strangled the intestine, and vomiting was produced when the dog swallowed.

A very remarkable fact, and worthy the attention of physiologists, is, that, by an injection of an emetic solution into the veins, vomiting was produced in animals from which the author had previously removed the abdominal muscles and diaphragm,—an experiment which indisputably proves that medicines of this class do not act upon the *abdominal* muscles, as we were led to conclude from the experiments of Mr. Magendie.

---

*Reponse de* G. Th. Marquais, *Chirurgien, au Memoire de* Mr. Magendie, *sur le Vomissement, et sur le Rapport fait a l'Institute, par* Messrs. Cuvier, Pinel, Humboldt, *et* Percy. pp. 17.

The Memoir of Mr. Magendie on Vomiting was scarcely brought to light, when Mr. Marquais entered the lists against him in the present pamphlet. Our author falls first upon Mr. Magendie, and then on the Commissioners of the Institute, and goes so far as to insinuate that both the Memoir and Report are the production of one pen.

Mr. Magendie, he observes, in the first place, is wrong in maintaining that towards the end of the 17th century physiologists and physicians were unanimous in considering vomiting as the effect of a convulsive contraction of the *stomach*, since Haller and Wepfer have spoken of the action of the diaphragm under these circumstances.

2. It is untrue that many authors of the fifteen last years of this century, and of one half of the following, was entirely passive in vomiting. Mr. Marquais, however, has *forgotten* to supply the proof.

3. Mr. Magendie is wrong in maintaining that Senac, Van Swieten, and Schwartz, have adopted the opinion of Chirac on the passive state of the stomach, as these authors contain many proofs in their writings to the contrary.

4. The author of the memoir said, Lieutaud combats the opinion of of Chirac by reasoning rather than by fact. Mr. Marquais finds in Lieutaud a fact which supports his reasoning.

5. Wepfer *did* distinguish the spasmodic or convulsive movements of the stomach from the permanent contractions which are produced by the action of certain chemical substances.

6. It is not a few experiments of Haller which have induced that physiologist to embrace his system of vomiting. They are the numerous observations made by Wepfer, and those of Fælix his pupil, made in his presence, and his own.

7. He accuses the Commissioners of partiality in disputing the experiments of Haller, while those of Mr. Magendie are considered well authenticated.

8. The first experiment of Mr. Magendie does not differ, in the author's opinion, from those of Bayle and Chirac.

9. Wepfer remarked *before* Magendie that the stomach filled with air during vomiting.

10. From the year 1668, Elcholz injected emetic substances into the veins of animals to excite vomiting.

11. Mr. Magendie, in his experiments, has demonstrated that it is not by the action on the stomach that the emetic occasions vomiting. M. Marquais agrees with him, but insists that the effect is produced by its operation on the œsophagus, and not on the diaphragm and abdominal muscles; and explains in the same manner how vomiting takes place when the pig's bladder is substituted for the stomach.

12. Mr. Magendie is not the first who made the section of the abdominal muscles, and preserved the peritoneum, to see if vomiting could be effected. The same experiment may be found, says M. Marquals, in a dissertation printed in 1698.

Other remarks of the same kind, and no doubt dictated by the same motive, are to be found in this pamphlet, but, out of compassion to the public, we shall cease to quote him. Whoever will take the pains to examine the Memoir on Vomiting, published in our 30th volume, will discover that the author of the paper under consideration principally labours to prove what is admitted by his opponent (if we can without insult to Mr. Magendie adopt the term). This gentleman does not take to himself the credit of entire originality, but certainly deserves that of having instituted a most interesting series of experiments, which put the question upon a firmer and more indestructible basis than it had ever possessed before. We know not who this M. Marquais is, but he has given us no proof either of the excellence of his temper or the soundness of his judgment. Mr. Magendie's reputation is too firmly established to be affected by petty quibbles, the consequences of which must of necessity revert to the snarler that gives them birth. Phædrus will supply M. Marquais with a parallel, and we trust he will be improved by the lesson; we refer him to the fable of the Viper and the File.

*Mordaciorem* qui *improbo dente* adpetit

Hoc argumento se describi sentiat, &c.

Cease, Viper, you bite against a file.

