# Errata

**Published:** 1815-01

**Authors:** 


					-P. 518, 1. 8, from ,bot-
torn, after the word following, arid asserted that the stomach.-

				

